# Plasma membrane aquaporins mediates vesicle stability in broccoli

**DOI:** 10.1371/journal.pone.0192422

**Published:** 2018-02-08

**Authors:** Maria del Carmen Martínez-Ballesta, Pablo García-Gomez, Lucía Yepes-Molina, Angel L. Guarnizo, José A. Teruel, Micaela Carvajal

**Affiliations:** 1 Aquaporin Group, Plant Nutrition Department, Centro de Edafología y Biología Aplicada del Segura (CEBAS-CSIC), Campus de Espinardo, Espinardo, Murcia, Spain; 2 Departamento de Bioquímica y Biología Molecular A, Facultad de Veterinaria, Universidad de Murcia, Espinardo, Murcia, Spain; Universita degli Studi di Bari Aldo Moro, ITALY

## Abstract

The use of *in vitro* membrane vesicles is attractive because of possible applications in therapies. Here we aimed to compare the stability and functionality of plasma membrane vesicles extracted from control and salt-treated broccoli. The impact of the amount of aquaporins was related to plasma membrane osmotic water permeability and the stability of protein secondary structure. Here, we describe for first time an increase in plant aquaporins acetylation under high salinity. Higher osmotic water permeability in NaCl vesicles has been related to higher acetylation, upregulation of aquaporins, and a more stable environment to thermal denaturation. Based on our findings, we propose that aquaporins play an important role in vesicle stability.

## Introduction

In recent years, high importance has been given to membrane vesicles due to the potential application as carriers in therapies. This vesicles coming from plasma membrane, called extracellular vesicles (EVs) has been reported as fundamental for intercellular communication [1]. The discoveries demonstrating that EVs are involved in a large number of metabolic regulations have driven further investigation of their precise role in each cellular and tissue functioning. In the same way, after isolation, the putative use as biomarkers, as vaccines, or as drug delivery are under investigation as for example the vesicles obtained from mouse mesenchymal stem cells that appeared to be effective in the suppression of hypoxia-induced inflammation [2]. However, the development of the EVs production from animal cells should be taken with care when *in vitro* animal culture cells are used due to their associated oncogenic potential [3].

In addition to being an important crop with high economic returns, Brassica crops might represent a source of agronomical by-products. For example, the marketable production of broccoli represents only 25% of its total biomass, the remaining by-product 75% (about 500.000 tons) consists of leaves and stems without commercial or economic value, involving additional agricultural waste management costs for farmers. Therefore, the derived by-products could be an important source of different biomolecules with industrial applications [4]. Recently, we found that chemical changes appeared in the plasma membrane of Brassica leaves and roots in relation abiotic stress resistance [5]. The response of the plasma membrane in lipid composition was related to the maintenance of protein functionality and permeability. Also, the higher amount of proteins was related to increases in ion and water transport that will determine a maintenance of cell homeostasis. Furthermore, *Brassica oleracea* isolated plasma membrane vesicles are more thermodynamically stable [5] and, thus, more suitable for storage due to its lipid and protein composition (therefore affecting its physical properties) than others from plants such as *Cakile maritima* or *Capsicum annum* [6]. Those results suggested that the lipid/protein ratio, degree of saturation, and sterol composition in relation to the application solution and target tissue for the design of proteoliposomes. This need is driving studies of membrane vesicles and their use for biotechnological applications [7]. The most important key of the EVs is the protein contents. Therefore, the study of their protein composition and functionality is the future area of research.

Aquaporins are membrane proteins that belong to the major intrinsic protein (MIP) family. Aquaporins appeared in all living organisms, in which they function mainly as water channels, but small neutral solutes (urea, silicon, boron, hydrogen peroxide) or gases (ammonia and carbon dioxide) are also transported. Aquaporins in plants have been classified into different subfamilies according to their sequences and cellular locations: the plasma membrane intrinsic proteins (PIPs), tonoplast intrinsic proteins (TIPs), NOD26-like intrinsic proteins (NIPs) and small, basic intrinsic proteins (SIPs), the GlpF-like intrinsic proteins (GIPs), hybrid intrinsic protein (HIP), and the uncategorized X intrinsic protein (XIP) [8].

The aquaporin selectivity to substrate was determined by the conserved NPA motifs and amino acid residues including the ar/R (aromatic/arginine) region. The conserved NPA box was responsible for water molecules selectivity [8], which resulted highly conserved in the subfamilies PIPs and TIPs. However, variable motifs have been observed in the NIP or SIP subfamilies determining preference for other substrates rather than for water [9]. In fact, NIPs have less water transport activity, but higher permeability to small organic molecules and mineral nutrients than PIPs and TIPs [10,11]. In addition to water PIPs also transport glycerol, CO_2_, H_2_O_2_ and urea. In TIPs subfamily the permeability of water was pointed out as their main function, but TIPs also allow the pass of glycerol, urea, and ammonia. While NIPs, SIPs have moderate water transport activity, XIPs work as multifunctional channels permeable to water, metalloids and ROS [12].

Aquaporins are transmembrane channels, assembled in tetramers with a molecular mass of 25 and 30 kDa per monomer [13]. The aquaporins provide to whole plant, but firstly to roots, to adapt to changeable environmental conditions altering permeability of membranes. Therefore, pharmacological and overexpressing/knockout aquaporins experiments have pointed the importance of this proteins in root water uptake [14–16]. The study of the contribution of each individual aquaporin (isoforms) to root water uptake and to its regulation is under study, but it has been reported in our previous works that in broccoli the level of transcripts was higher under salinity stress conditions [17].

In 2013, aquaporin-reconstituted proteoliposomes were designed and produced for water purification filters. From these preliminary nanofiltration results, these polymerized vesicles were shown to be stable under pressure and stirring shear [18]. Therefore, in addition to facilitating water transport, aquaporins could be key players in membrane stabilization *in vitro*. Membrane vesicles from Brassica species, which are rich in aquaporins, have been extracted using a patented method (PCT/ES2012/070366) [19] for use in industrial applications. However, little is known about how molecular interactions (between lipids and proteins) affect the life-storage time of plants. Therefore, the study of stable natural Brassica vesicles enriched in proteins (aquaporins) will be useful for several applications.

Here, we investigated the effect of increased aquaporin content on the stability of broccoli extracted vesicles. Also, the pool of aquaporins was related to the osmotic water permeability of the vesicles and the stability of the plasma membrane protein secondary structure. PIP acetylation was identified under high salinity and the potential role of this modification as a protective mechanism against protein degradation is discussed.

## Material and methods

### Plant growth

Seeds of broccoli (*Brassica oleracea* L. var. Italica cv Naxos) were germinated and cultivated as previously reported in Muries et al. [8] with some modifications. The pre-hydration with de-ionized water in continuous aeration was carried out for 24 h. The seeds were germinated in vermiculite in the dark at 28°C for two days and the sprouts were then transferred and cultivated in hydroponic solution in a controlled-environment chamber. After two weeks of growth, an osmotic shock (12 dS m^-1^) as 100 mM NaCl treatment was applied to half of the plants. The roots were harvested for plasma membrane isolation after another two weeks of growth.

### Plasma membrane isolation

Root plasma membranes were purified using the two-phase aqueous polymer technique first described by Larsson et al. [20] and modified by Casado-Vela et al. [21]. The purity of the plasma membrane preparation was estimated by measuring the enzymatic activities of the plasma membrane and other organelles [22].

### Size of vesicles

The average size of the vesicles was checked using light-scattering technology; through intensity measurements with a Malvern ZetaSizer Nano XL machine (Malvern Instruments Ltd., Orsay, France), as previously described [23]. This allowed the analysis of particles with sizes ranging from 1 nm to 3 μm.

### Total protein degradation

The protein concentration was assayed by the Bradford method [24] using bovine serum albumin as standard. To test resistance to degradation, the vesicles were stored at 4°C for 7 days. The concentration of proteins was measured every day after centrifugation at 100,000 *g* for 30 min to ensure precipitation of integral vesicles.

### Stopped-flow light scattering

The osmotic water permeability (*P*_*f*_) was measured by the velocity of the volume adjustment of the membrane vesicles after changing the osmotic potential of the surrounding media. The volume of the vesicles was followed by 90° light scattering at λex = 515 nm. Measurements were carried out at 20°C in a PiStar-180 Spectrometer (Applied Photophysics, Leatherhead, UK), as described previously [25]. The vesicles were stored at 4°C for 4 days to test their resistance to degradation. Pf was measured every day.

### Gel electrophoresis

A volume of the plasma membrane-enriched preparation corresponding to 50 *μ*g was resolved on a 12% SDS-PAGE gel using a Mini-PROTEAN gel electrophoresis system (Bio-Rad) at 100 V/h. The gel was stained with Bio-Safe Coomassie G-250 (Bio-Rad) for 1 h.

### Endoprotease in-gel digestion and protein identification

The gel bands corresponding to PIPs (monomers and dimers) from three SDS-PAGE gel lanes were excised from the gel and pooled in a single vial for protein identification by HPLC-MS/MS analysis, as it was previously described [12]. However, in this work, the results of root membranes from control plants were compared with NaCl treated plants.

### Gel electrophoresis and immunoblotting

Plasma membrane from the roots of broccoli plants was isolated as described above. Protein (10 μg per lane) was loaded for 12% sodium dodecyl sulfate-polyacrylamide gel electrophoresis (SDS-PAGE) as described previously [17]. The *Bo*PIP2 antibody was synthesized from the peptides sequenced by high-resolution mass spectrometry [21]. The antibody was raised against the 42 N-terminal residues of PIP1;1 from *A*. *thaliana* and the acetylated 14 N-terminus residues of *B*. *oleracea* var. Italica PIP1 (PIP1;1, PIP1;2 PIP1;3 and PIP1;4). Goat anti-rabbit IgG coupled to horseradish peroxidase was used as the secondary antibody (dilution 1:20 000). A chemiluminescent signal was developed using the West-Pico Super Signal substrate (Pierce, Rockford, IL).

### Infrared spectroscopy

The sample absorbance in the mid-IR region (400–4000 cm^-1^) was measured in a 6700 Fourier-transform infrared spectrometer (FTIR) (Madison, WI) equipped with a sample holder thermostatized by a Peltier device (Proteus system from Nicolet). Plasma membranes were centrifuged at 100,000 *g* for 35min at 4°C. The pellet was washed and centrifuged twice with phosphate buffer 5 mM, 0.25 M sucrose (pH 6.5) prepared in ^2^H_2_O and kept at 4°C overnight before use to allow a complete H_2_O-^2^H_2_O exchange. Twenty microliter pellets (~30 mg protein ml^-1^) were placed between two CaF_2_ windows separated by 50 μmTeflon spacers and transferred to a Symta cell mount. The equipment was continuously purged with dry air to minimize the contribution peaks of atmospheric water vapor. For each spectrum, a total of 250 interferograms were collected with a nominal resolution of 2 cm^-1^. Spectra were collected at approximately 2°C intervals, allowing 5 min equilibration time at each temperature. The ^2^H_2_O buffer spectra taken at the same temperature were subtracted interactively using GRAMS/32 software (Galactic Industries, Salem, MA, USA), as described previously [26,27].

The secondary structure of the protein was quantified by curve-fitting of 8 components to the amide I band using GRAMS/32 software (Galactic Industries, Salem, MA, USA).

The FTIR spectrum of proteins has a number of characteristic. Amide bands, which is due to the vibration of the atoms involved in the peptide bond. Amide I and II absorption bands are two major bands of the protein infrared spectrum in the region between 1800 and 1500 cm^-1^. The Amide I band (1700–1600 cm^-1^) was obtained mainly from the C = O stretching vibration (70–85%) having less contributions of the out-of-phase C-N stretching vibration (10–20%), and it is the most intense band in proteins [28]. The Amide I band can be analyzed by different procedures to obtain information about the presence of different secondary structures in the protein. In this work, the Amide I band has been decomposed into its constituents by curve-fitting to quantitatively estimate the area of each component representing a type of secondary structure [29]. A total of eight assignments to secondary structures has been made to properly decompose the Amide I band. These eight components were centered at 1692 cm^-1^ (β-turns), 1680 cm^-1^ (β-sheet), 1669 cm^-1^ (β-turns), 1656 cm^-1^ (α-helix), 1644 cm^-1^ (unordered), 1632 cm^-1^ (β-sheet), 1625 cm^-1^ (unfolded), and 1615 cm^-1^. A component with the lowest contribution, previously assigned to Tyr side chain vibrations [30], was not been included in the analysis.

### Data analysis

The statistical analyses were carried out using SPSS Release 18 for Windows. Tukey’s HSD test at the P ≤ 0.05 was chosen to determine significant differences between treatments. Small letters on top of bars point the significant differences between treatments.

## Results

**Fig 1:**
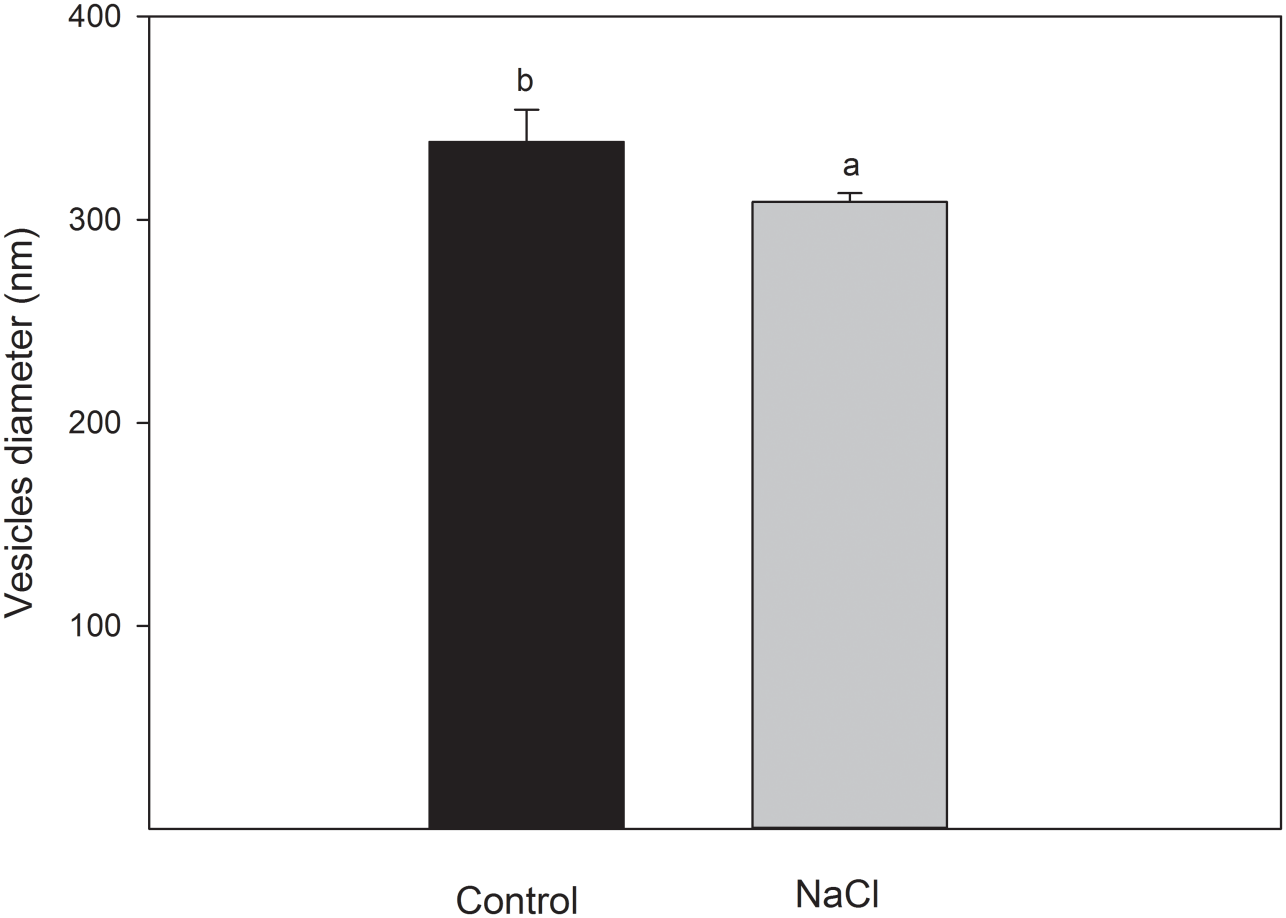
The average size of plasma membrane vesicles diameter from roots of broccoli control and 100 mM NaCl-treated plants. Data are means ± SE (n = 5, n = individual plasma membrane extraction with a protein concentration of 1 mg ml^−1^).

The determination of the size of the root vesicles revealed a significant difference between controls and roots obtained from NaCl treated plants (Fig 1). The size of vesicles from control plants was higher than from NaCl treated plants (340 nm vs. 309 nm). Also, the variability in the size of the control vesicles was higher than vesicles from the NaCl treated plants.

**Fig 2:**
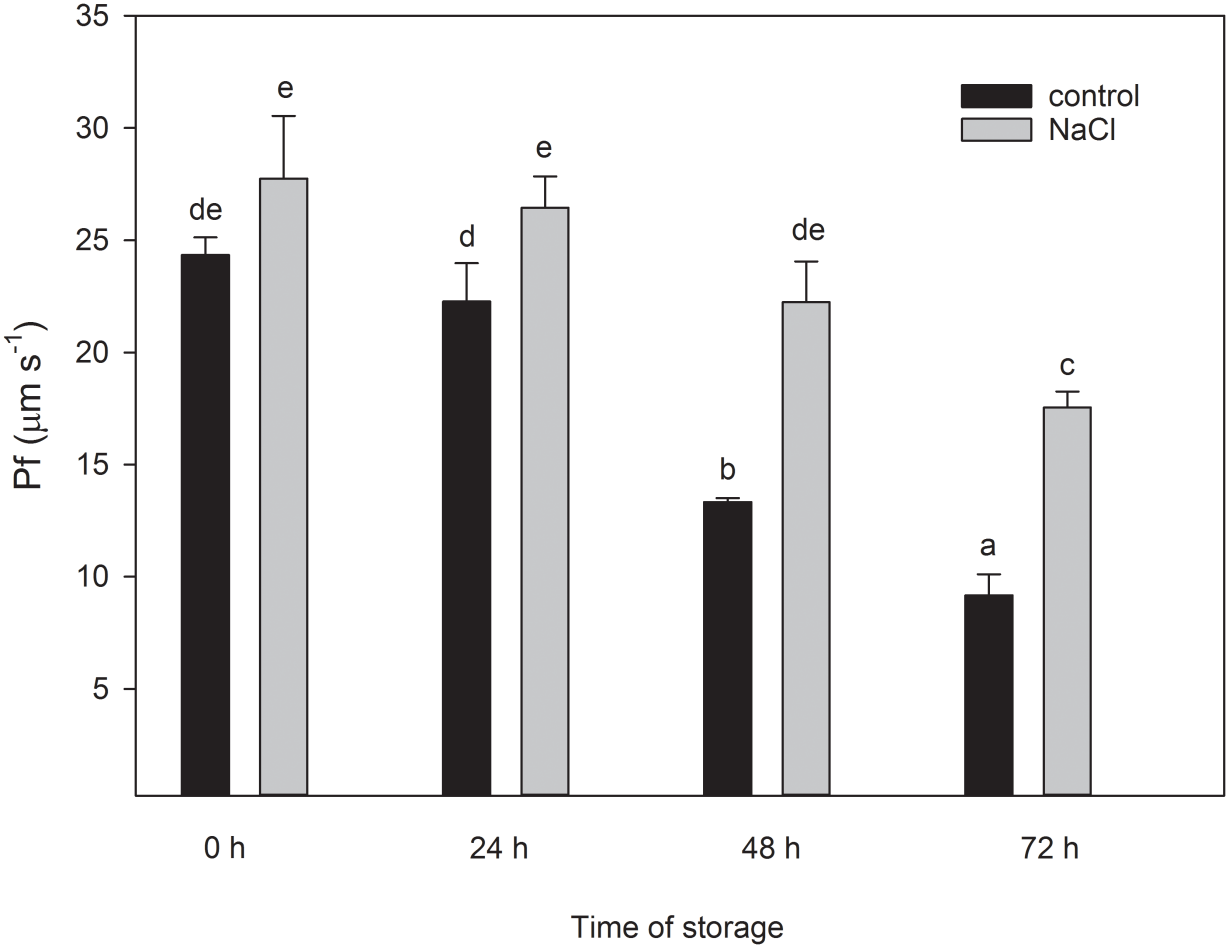
Time course (days) of the osmotic water permeability (P_f_) of root vesicles at 4°C isolated from control and 100 mM NaCl-treated plants. Data are means ± SE (n = 30).

To determine the integrity of the vesicles, the capacity for water transport of isolated vesicles form plasma membranes of broccoli roots was measured after 0 to 3 days of storage at 4°C. For that, osmotic water permeability values (*P*_*f*_) were measured for purified plasma membrane vesicles, using stopped-flow light scattering [31,32]. We found that there was a slight increase with the NaCl treatment on the P_f_ values, compared to the control at 0 h (Fig 2). The highest *P*_*f*_ was obtained in the NaCl treatment samples in all times of measurement. The *P*_*f*_ of the NaCl treated samples becomes significantly different from the control after 1 d, and this difference becomes progressively greater throughout the storage period.

**Fig 3:**
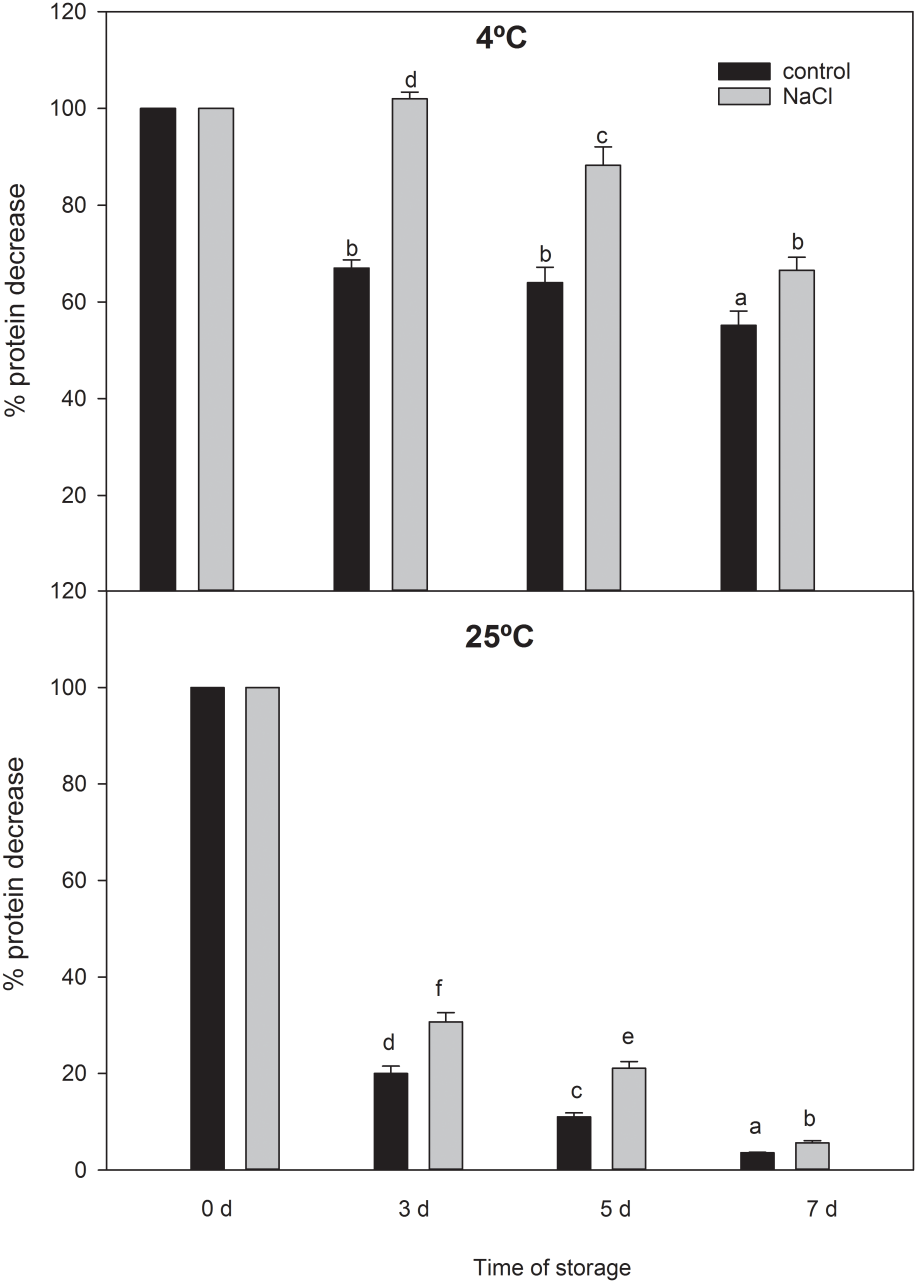
Protein degradation during 7d, at 4°C and 25°C, of broccoli root vesicles isolated from control and 100 mM NaCl-treated plants. Data are means ± SE (n = 30).

The concentration of proteins in the vesicles was also measured after 0 to 7 days of storage at either 4°C or 25°C. The protein stability was expressed as the percentage relative to the initial protein concentration (Fig 3). At 4°C, a strong decrease (20%) was observed in root control vesicles after 3 d and 5 d. After 7 days, this decrease reached almost 50%. However, the NaCl root vesicles showed no significant differences after 3 d compared to their concentration at the initial time. At 25°C, a high decrease was observed for both control and NaCl after 3 days of storage. However, again the decrease was higher in control vesicles than in the NaCl treated vesicles for all time points.

**Fig 4:**
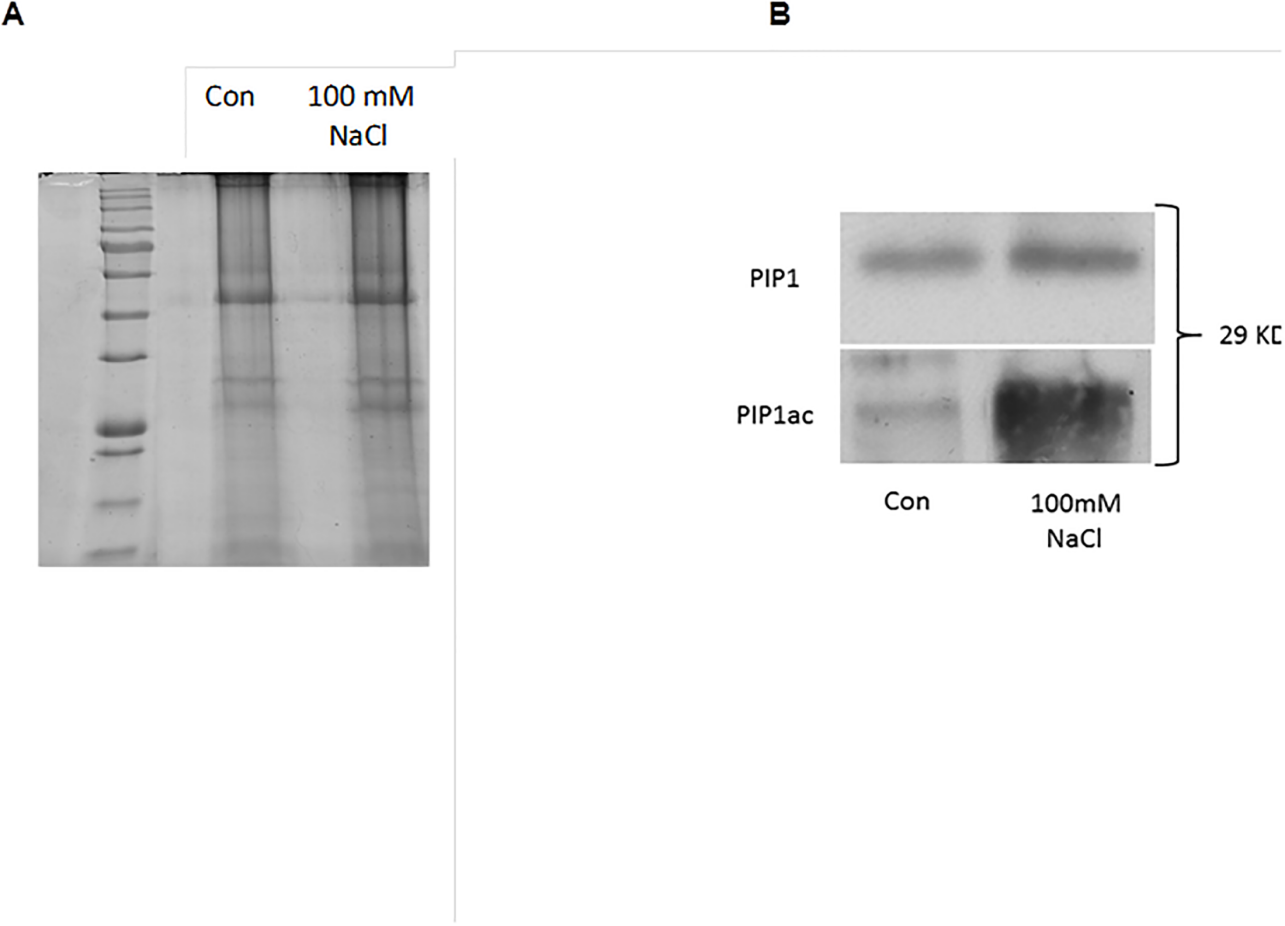
A) Purified plasma membrane (PM) proteins analysed in the roots of control and 100 mM NaCl-treated broccoli plants by 12% SDS-PAGE and stained with Coomassie Brilliant Blue (B) Immunodetection of PIP1 and PIP1 acetylated homologues in the root PM of control broccoli plants and plants treated with 100 mM NaCl. Total PM was separated by SDS-PAGE and probed with an antibody against *At*PIP1;1 and *Bo*PIP1 acetylated. Equal amounts of protein (10 μg) were loaded in each lane. Mean values are shown (n = 3).

Fig 4A shows the SDS-PAGE gel of plasma membrane proteins of control and NaCl treated plants. Note the presence of a lower band of ca. 30 kDa, corresponding to the monomeric form of PIPs, and an upper band of 60 kDa, corresponding to dimeric forms of PIPs. It can be observed that the bands corresponding to the plasma membrane extraction from the NaCl treated plants were denser than the control. We also introduced the use of Glu-C based on the observation that AtPIPs include a relatively high number of Glu and Asp residues. The peptides derived after each endoprotease digestion were analyzed by HPLC-MS/MS. As shown in Table 1, similar aquaporin isoforms (Table 1) previously identified by Muries et al. [21] were found after NCBI and SwissProt database searches. In addition to the PIPs, two TIPs (tonoplast intrinsic protein), TIP 1;2 and TIP 2;3 were identified, which has also been observed in previous proteomic studies of *Arabidopsis* leaf [33] and root [34] plasma membranes. Also, in our samples, the NIP1;2, isoform, a plasma membrane-localized member, was identified in the roots. Other proteins related to cell wall organization, signal transduction and secretory pathway, transporters, protein regulation, and cell growth and differentiation were described (Table 1).

**Table 1:** Proteins identified in the 30 kDa and 60 kDa bands of the plasma membrane proteins fraction of control and 100 mM NaCl-treated broccoli roots, roots after database search, where AAs are the number of aminoacids, coverage, the percentage of the protein sequence covered by identified peptides and peptides the number of distinct peptide sequences in the protein group.

	NCBI or Swiss Prot Accession	AAs	MW(Da)	Description	Coverage	Peptides
Cell Wall organization	gi15225712	757	84206,29	ATCSLB02 Cellulose synthase-like B2 transferase	1,72	1
Signal transduction- Secretory pathway	gi18399786	440	49819,55	ATPDIL5-2 (PDI-LIKE 5–2); thiol-disulfide exchange intermediate	3,18	5
	gi18418600	512	56781,73	protein kinase family protein	8,01	11
	gi238621682	422	47615,14	protein kinase-related	9,72	21
	gi223549702	273	30797,22	receptor protein kinase	10,62	6
	gi229851673	302	32161,21	universal stress protein UspA-like protein	5,30	5
	gi79327256	223	25207,05	SYP132 (syntaxin 132); SNAP receptor	7,62	1
	gi146150661	208	22812,90	resistance-gene-interacting protein	8,65	1
	A5DXI9	362	41667,20	Actin cytoskeleton-regulatory complex protein END3	3,87	
	Q8RY89	769	87347,74	Phosphatidylinositol-4-phosphate 5-kinase 8	2,60	1
	gi222476495	227	24634,86	translation elongation factor 1 alpha	4,85	17
	P0C7R4	658	73842,98	Pentatricopeptide repeat-containing protein	1,98	5
	A3LPW2	511	59735,82	Protein FYV10	1,96	1
Transporter						
	gi15228095	285	30433,68	PIP 2;2	6,32	1
	gi7209556	286	30523,99	PIP 1;2	6,29	7
	gi7209560	283	30100,45	PIP 2;2	6,36	1
	P30302	285	30409,69	PIP2;3	3,86	16
	gi62319319	67	7221,73	PIP 2;1	16,42	7
	gi15223438	286	30612,94	PIP1;3	5,24	1
	Q06611	286	30578,01	PIP1;2	9,79	269
	gi42572785	219	23312,92	PIP 1;4	5,02	2
	P61837	286	30668,98	PIP1;1	16,08	351
	gi332643653	253	25848.9	TIP1;2	5,44	1
	gi15238100	250	25245.6	TIP2;3	5,08	1
	gi32363340	294	31269.5	NIP1;2	7,66	1
	gi9858170	453	48693,44	plasma membrane H+-ATPase	7,28	2
	gi758250	951	104376,93	H(+)-transporting ATPase	1,47	1
	gi13366070	174	19094,94	plasma membrane H+-ATPase	8,05	1
	gi62321152	288	31550,48	plasma membrane-type calcium ATPase	5,90	2
	gi2911803	69	7093,90	plasma membrane H+-ATPase	24,64	2
	gi9971067	588	64826,99	Nitrate transporter	2,89	1
	O81108	1014	110368,25	Calcium-transporting ATPase 2	1,68	2
	P23980	704	77990,52	Plasma membrane ATPase 2	1,99	3
	P92935	618	67485,82	ADP,ATP carrier protein 2	1,94	7
	P19456	948	104334,99	ATPase 2, plasma membrane-type	4,64	25
	Q42556	954	105141,76	ATPase 9, plasma membrane-type	2,41	7
	P46032	585	64379,59	Peptide transporter PTR2	2,22	1
	P83970	951	104618,27	Plasma membrane ATPase	2,42	3
	P23586	522	57572,98	Sugar transport protein 1	2,30	
	Q9SZN1	487	54270,67	V-type proton ATPase subunit B2	2,87	1
	O82226	747	85386,45	Cyclic nucleotide-gated ion channel 6	1,87	9
Cell growth and differentiation	gi8809602	159	17510,81	ras-related small GTP-binding protein-like	10,69	2
	Q39571	203	22584,48	GTP-binding protein YPTC1	7,88	1

The quantification of acetylated PIP1 aquaporins was carried out using Western blot analysis. Only one band corresponding to 29 kDa (aquaporin monomer) was detected with PIP1ac antibody in control and NaCl treated plants (Fig 4B). However, the immunostaining intensity corresponding to PIP1 was higher in the 100 mM NaCl- treated plants than in control plants. Interestingly an strong increase in the acetylated PIP1 content in the salinity treatment was observed, while under control conditions PIP acetylation was quite low.

**Fig 5:**
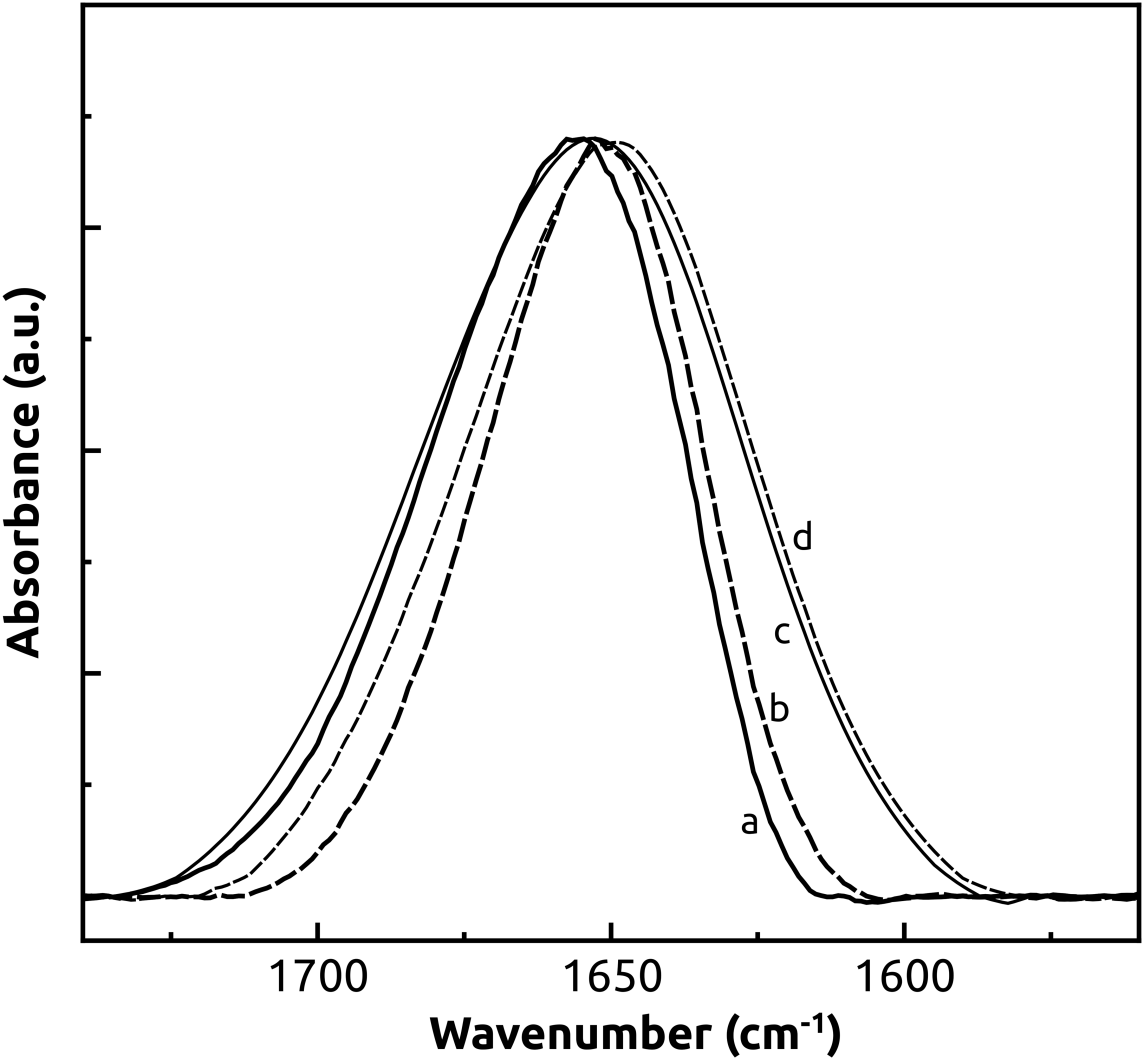
FTIR spectra of the Amide I band region of plasma membrane preparations in D_2_O solutions. Control plasma membrane at 24°C (thick solid line, a) and 70°C (thick dashed line, b), and plasma membrane treated with 50 mM NaCl at 24°C (thin solid line, c) and 70°C (thin dashed line, d).

To address the effect of the saline treatment on the plasma membrane integrity, the infrared spectra of the samples were recorded at increasing temperatures from 24°C to 70°C to promote thermal denaturation of the proteins. The effect of heating, as well as the effect of salt treatment with 100 mM NaCl on the shape of the Amide I band infrared spectra, is shown in Fig 5. The frequency and shape of the Amide I band is determined by the backbone conformation and the hydrogen bonding pattern, and thus, it appears to be sensitive to the secondary structure of the protein. In both control and salt-treated samples, the effect of heating up to 70°C was a band narrowing at higher wavenumbers, while the effect of salt treatment was mainly to broaden the Amide I band at lower wavenumbers (Fig 5).

**Fig 6:**
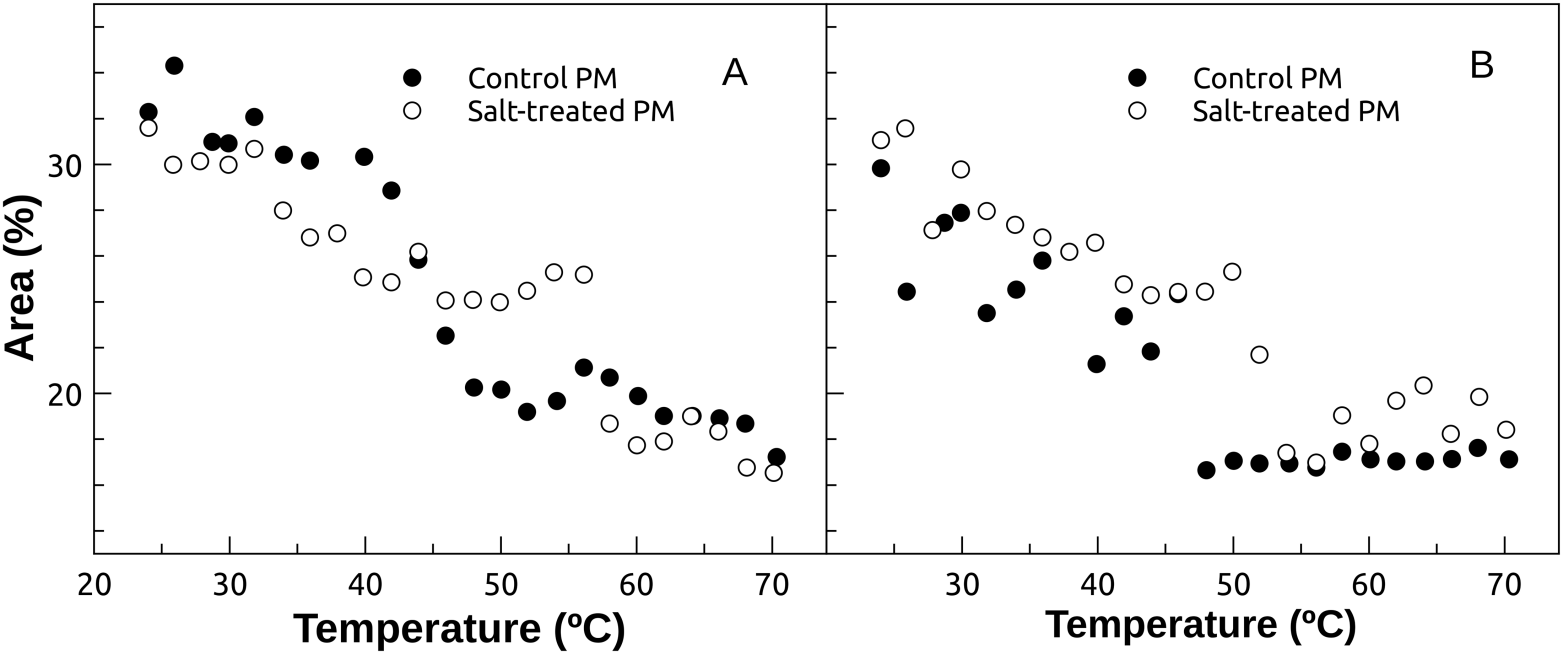
β-sheet and β-turns secondary structures. β-sheet structure corresponds to bands centered at 1680 cm^-1^ and 1632 cm^-1^ (A), and β-turns structure to bands centered at 1692 cm^-1^ and 1669 cm^-1^ (B). Control and salt-treated plasma membrane samples are shown in closed and open circles, respectively.

The Amide I bands were decomposed by curve-fitting to ascribe the observed band shape changes to secondary structure alterations of the pool of proteins in the plasma membrane fraction. The area of each component was calculated to determine the percentages of change of the secondary structures. This procedure was performed with control and salt-treated plasma membrane samples from 24 to 70°C at approximately 2°C intervals. Fig 6A shows the results obtained for the bands centered at 1680 cm^-1^ and 1632 cm^-1^ corresponding to the β-sheet structure. We detected a thermal transition in a β-sheet structure in the control plasma membrane sample occurring at about 40°C. In contrast, this transition was observed at a higher temperature in the salt-treated sample (about 56°C). A similar behavior was obtained for β-turns structure (Fig 6B). A thermal transition was observed at a lower temperature in the control sample (about 42°C) compared to the salt-treated sample (about 50°C).

**Fig 7:**
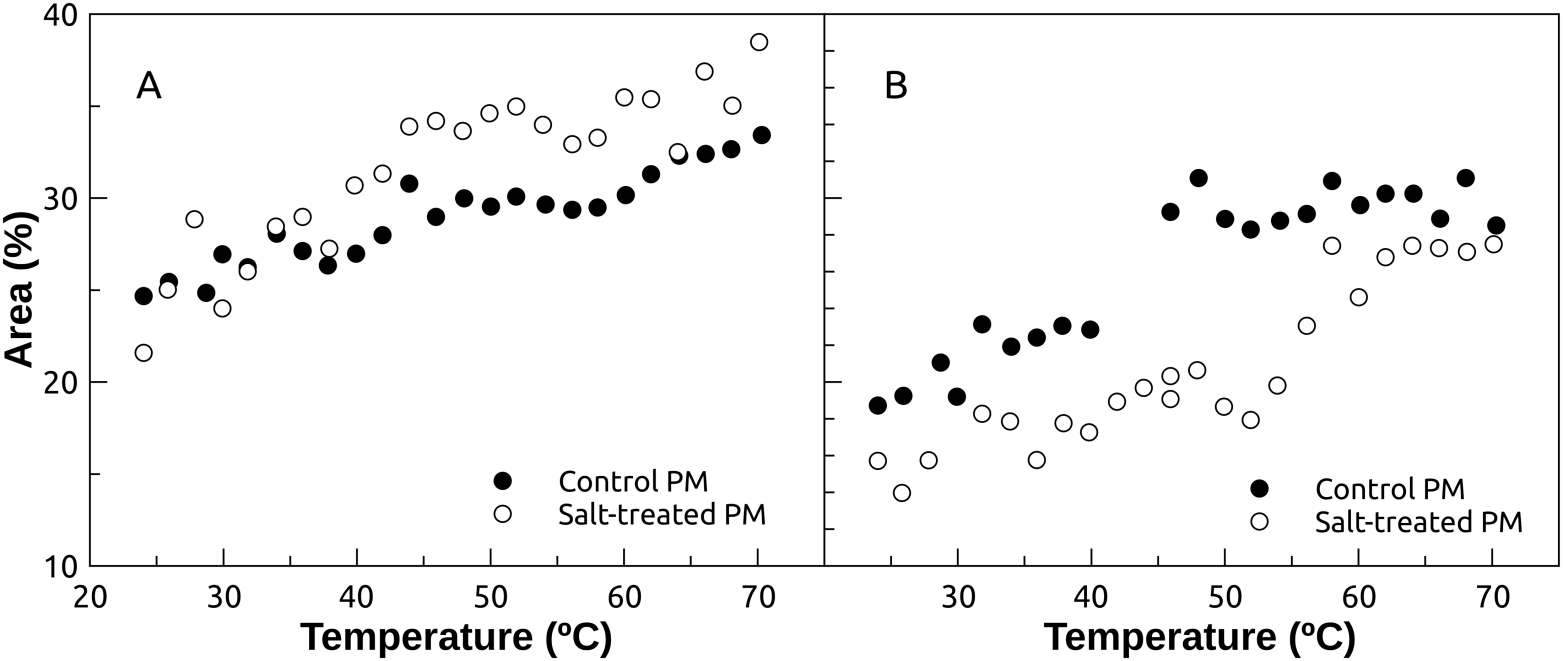
Unordered, unfolded and α-helix secondary structures. (A) Unordered and unfolded structures corresponding to bands centered at 1644 cm^-1^ and 1625 cm^-1^, respectively. (B) α-helix secondary structure corresponding to the band at 1655 cm^-1^. Control and salt-treated plasma membrane samples are shown in closed and open circles, respectively.

In Fig 7A, the structures involved in protein denaturation (unordered, unfolded, and possibly aggregation) are depicted corresponding to bands centered at 1644 cm^-1^ and 1625 cm^-1^. However, we did not detect a thermal transition, but rather a gradual increase of these structures for both control and NaCl treated plasma membrane vesicles. In Fig 7B, the α-helix secondary structure corresponding to the band at 1655 cm^-1^ can be seen. Also, an increase in α-helix structure content is observed for both control and NaCl.

**Fig 8:**
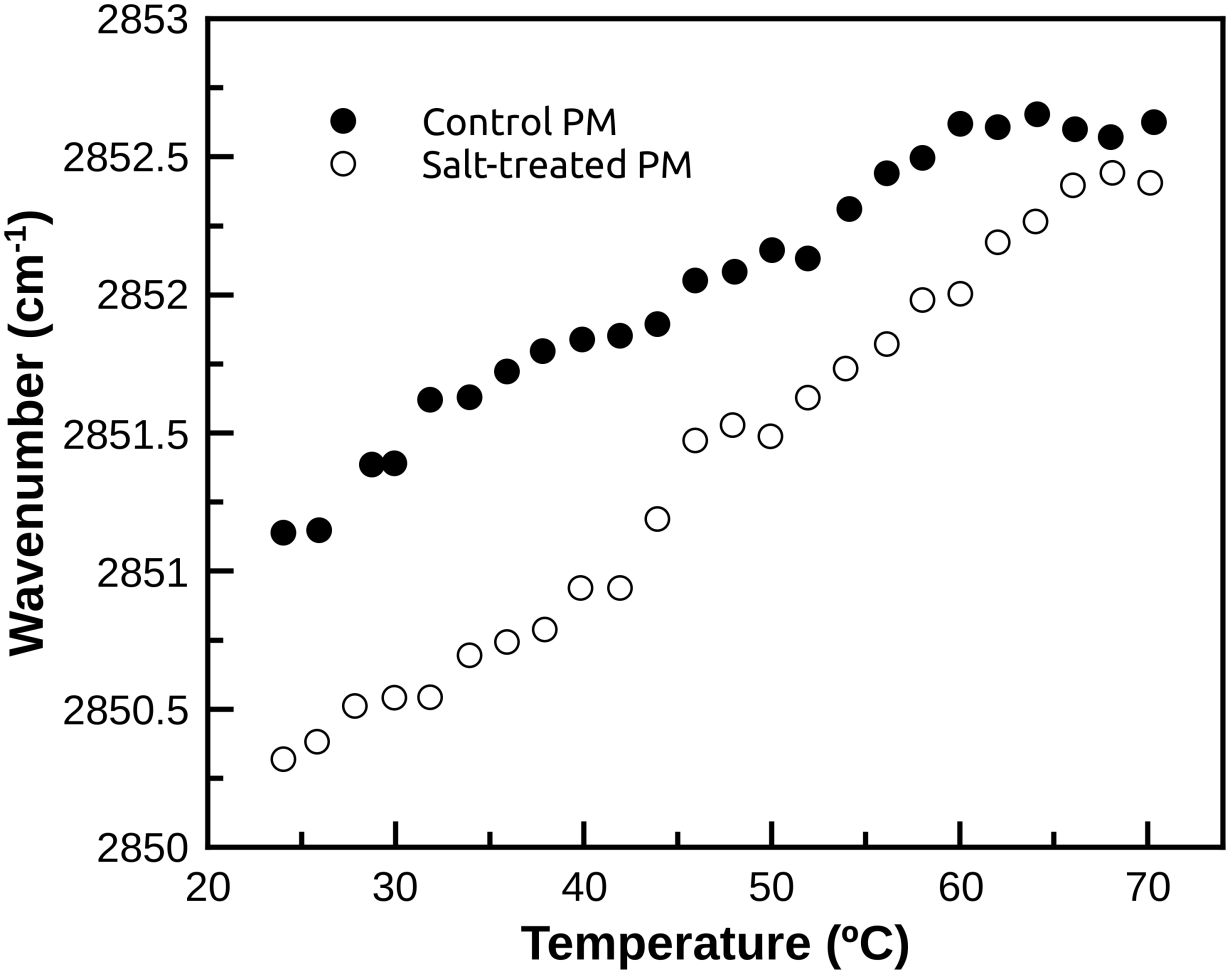
CH_2_ symmetric stretching. Changes of the absorption maximum of the CH_2_ symmetric stretching band of control (closed circles) and salt-treated (open circles) plasma membrane samples.

Fig 8 shows the absorption maximum of the CH_2_ symmetric stretching band determined in the 24–70°C temperature range. It can be seen that in the salt-treated plasma membrane sample there is a displacement of the absorption maximum towards a lower wave number, indicating that at 24°C the lipid membrane is in a more ordered state as compared to the control sample. In both cases, a gradual increase in wavenumber is observed with temperature (Fig 8). Higher temperature involves higher molecular kinetic energy and, consequently, higher molecular motion contributing to a trans/gauche ratio decrease and a more unordered lipid membrane.

## Discussion

Under salt stress, salt-resistant plants adapt their metabolism for maintaining growth [35]. Broccoli plants have been investigated in these terms, and the results give a higher aquaporin transduction in NaCl treated plants [22]. Also, in other experiments, results have shown that broccoli plants under high salinity stress (80 mM NaCl) markedly modify the plasma membrane lipid composition and induce a significant accumulation of PIP1 and PIP2 homologs [36]. Interestingly, when broccoli plants were exposed to salinity stress (100 mM) for 15 d, the transcript level of BoPIP2;2 and BoPIP2;3 was increased in roots of the Naxos cultivar [17]. In our plasma membrane extractions, the protein yield was always double in NaCl plants compared to the control (data not shown). A transcriptional aquaporin control has contributed to the amount of PIPs in the plasma membrane. However, although the diameter of the plasma membrane vesicles obtained from NaCl-treated plants was lower than that those obtained from control plants [36], the relationship between size and yield cannot be established.

To characterize the stability of aquaporins, the osmotic water permeability (*P*_*f*_) of vesicles extracted from broccoli roots was measured using stopped-flow light scattering [31]. In our results, although there were no significant differences between the *P*_*f*_ of plasma membrane vesicles of control and NaCl treated plants at initial time, the *P*_*f*_ was more stable during storage for the NaCl vesicles. Therefore, the size effect is discharged. The *P*_*f*_ measurement can also be related to the aquaporin functionality [31,37], but not with the aquaporins presence in broccoli [36]. Inhibitory effect on aquaporins in vesicles has been reported to be related to protons and divalent cations [38,39]. However, the fact that a higher decrease in *Pf* was observed after storage in control vesicles than in vesicles derived from salt-treated plants could be related to the pool of proteins and, therefore, with membrane integrity, rather than with functionality reduction.

Thus, the relative lipids abundance and differences in their selectivity can determine the ability of a local lipid environment to enclose aquaporins, thereby providing a fine mechanism to adjust and regulate protein structure and function [40]. In an *in vitro* study, it has been proposed that the AqpZ aquaporin was stabilized by different lipids exerting resistance to unfolding with a direct effect on the protein properties [41]. In our previous report, we demonstrated that changes in the lipid/protein ratio of the plasma membrane in different Brassica species conferred distinct physical properties to the lipid bilayer determining a salt tolerance rank related to the membrane protein presence [5].

While N-terminal acetylation has been studied in soluble mammalian and yeast proteins, the acetylation in the methionine of PIP1 subfamily has been described in much lesser extension for plant aquaporins [21,42,43]. The biological significance of this co- or post-translational modification has been determined in several protein studies [44], and different functions such as membrane targeting, protein-protein interactions and changes in proteostasis have been assigned. The most prominent effect of N-terminal acetylation relates to protein stability, increasing the half-life of proteins, and conferring resistance to degradative processes in the cell [45]. In AQP0, N-terminal acetylation provided a protective mechanism against N-terminal truncation [46]. Increasing acetylation in vesicles from salt-treated plants could be related to protein stability in broccoli, although the relation between acetylation and the presence of aquaporins in the membrane deserves further attention.

The structural amount of aquaporins in plasma membrane vesicles only can be determined relative to the rest of the plasma membrane proteins. In fact, the first quantitative results about the PIPs provide a quantification of 1% of PIP1 subfamily [47] and 3% of the remaining PIPs [48] among the plasma membrane (PM) proteins from *A*. *thaliana*. Measuring PIP relative abundance by SDS-PAGE stained densitometry, it was previously reported that they could constitute 15% of spinach leaves purified plasma membrane [49]. Later, although results confirmed that PIPs are major proteins of the plant plasma membrane, the difficulties with detection of ^15^N labeling or because they were only identified with peptides shared by other proteins do not allow the accurate in quantification [50].

In addition to the previously characterized aquaporin isoforms in the broccoli plasma membrane [21], other proteins were identified. Understanding PIP regulatory mechanisms and their role in the adjustment of plant water status by their interaction with other membrane proteins is an alternative approach [51]. Thus, aquaporins interactome studies are emerging, and some reports have demonstrated a PIP subfamily interactome controlling many physiological processes in the plant cell, including osmoregulation under high osmotic stress, such as under a high salinity. Among the identified proteins in broccoli, cell wall cellulose protein was found. It has been suggested that the regulation of cell wall cellulose synthesis has an important role in polysaccharide metabolism and adaptations of broccoli plants to salt stress [52]. Knockdown mutants for the cellulose synthase gene showed overexpressed *BoPIP2;2* and *BoPIP2;3* aquaporins compared to the WT plants, pointing out that these two isoforms enhanced the salt tolerance of mutant plants. However, interferences between cellulose synthase and aquaporins have not yet been addressed experimentally.

Proteins involved in the signal transduction secretory pathway were also identified. The reversible dithiol/disulfide transitions were carried out using the thiol-disulfide exchanger that regulates the activity of proteins by redox modulation in important physiological processes [53]. It has been postulated that aquaporins are redox-regulated membrane components. The changes in the ratio between dithiol and disulfide bonds modified water osmotic permeability, *P*_*f*_, and the authors assumed that redox transitions in the loop C were probably accompanied by changes in aquaporin conformation from the closed to the open pore state [54].

The infrared results showed that, in both cases (control and NaCl), a decrease of β-sheet and β-turns secondary structures were found with increasing temperature caused by a disruption of the protein secondary structure leading to protein denaturation. However, it could be concluded that saline treatment would develop a more stable environment to thermal denaturation, probably by changing the lipid composition of the plasma membrane as a response to the saline stress as it has been previously reported [6]. However, a number of proteins in vesicles have been directly related to higher stability as produced by phosphate transport induced by Pi starvation in *Arabidopsis* [55]. Also, in the chemical stability, temperatures stability and pressure stability of artificial membrane were related to aquaporins incorporation [56].

According to the proteins in our membranes, in general terms, thermal denaturation involved an increase in unordered and unfolded structures with a concomitant decrease in, β-sheet and β-turns structures. It has been proposed that thermally induced protein aggregation process comprise the formation of an intermolecular β-sheet structure, which appears at 1620–1625 cm^-1^ [57,58]. Thus, the assignment of the unfolded structure could also include aggregated states. In both cases, a decrease of β-sheet and β-turns secondary structures was found with increasing temperature caused by a disruption of the protein structure leading to protein denaturation. However, it was observed that the saline treatment would develop a more stable environment to thermal denaturation. This could be due to the type of proteins expressed or by the interactions with the lipid composition of the plasma membrane [7].

In membrane proteins, the α-helix is the more common secondary structure of the transmembrane segments. Hence, α-helix content would come from the contribution from globular and intramembranous segments. An increase in the α-helix structure is clearly not expected upon protein thermal denaturation. This anomalous behavior could point out the presence of α-helices deeply buried in the plasma membrane, where the accessibility of D_2_O would be diminished, inhibiting deuterium exchange. In H_2_O α-helix cannot be properly resolved since α-helix band overlaps with the signal obtained from the unordered structure, which appears at 1642–1657 cm^-1^, but these two bands can be resolved in D_2_O because the unordered band is displaced towards lower wavenumbers [28,59,60]. Unfortunately, incomplete deuterium exchange would complicate the interpretation of the 1642–1657 cm^-1^ region and could lead to unexpected behavior, as shown in Fig 7B.

Also, previous studies have reported that high salt concentrations in the growing medium increased the plasma membrane sterol content [6,36] with the concomitant increase in the membrane order providing a more rigid membrane [61] and higher efficiency in regulating water permeability [6,62]. In our results, information on the properties of the lipid membrane can be obtained from the vibrational stretching mode of the CH_2_ group in the region of 3100–2800 cm^-1^. The CH_2_ antisymmetric and symmetric stretching bands at about 2929 cm^-1^ and 2850 cm^-1^, respectively, are the most characteristic bands in the lipid membrane infrared spectra. Both bands appear to be sensitive to the conformation of the lipid membrane. Changes in the absorption maximum frequency respond to changes of the trans/gauche ratio in the lipid acyl chains. In particular, the symmetric stretching band is the most useful band to analyze the gauche conformers content of the lipid acyl chains, since the antisymmetric band might be a result of the overlapping with other vibrational modes, such as CH_3_ stretching. It has been described that a decrease in the wavenumber maximum is produced by a decrease in the lipid trans/gauche conformers ratio which in turns causes a decrease of the order of the lipid membrane moiety [63]. Also, membrane vesicles stability has been related to lipid peroxidative properties of the membranes or the protective effect of bioactive compounds [64]. Hence, the better protective effect extract against membrane disruption was related to the better anti-oxidant properties.

The results obtained according to CH_2_ symmetric stretching band showing that membranes from NaCl treated plants were more ordered than control is in agreement with previously reported results suggesting that hydric stress causes a modification of the lipid membrane composition making a more rigid membrane helping the cell to maintain its hydric state upon adverse hydric conditions [6,65,66].

## Conclusions

Here we detected a decrease in the size of vesicles extracted from broccoli roots given salinity treatments compared to non-treated controls. Although this finding cannot be related to our other findings, which indicates higher stability, it is in line with the observed low fluidity and low unsaturation that could provide a reduced area per lipid and, therefore, a reduced surface area. The size of vesicles is important factor for the studies of drug carriage and delivery [67], but not for their stability.

Therefore, treatments of NaCl to broccoli plants conferred two effects: 1) modification of lipids for a more impermeable barrier; and 2) an increase in the acetylation of aquaporins for high water permeability. Although these findings might seem contradictory, they indicate that water transport through aquaporins is highly regulated, allowing the plant to achieve precise regulation of water transport under stress conditions. The NaCl-induced biochemical changes shown in this work provide the vesicles with higher *in vitro* stability. In fact, the stability could be related to the interactions of multiple factors, such as lipid changes, protein increase, and protein physical properties. In this work, the type and amount of proteins have been demonstrated to play a determinant role in the stability of the vesicles, with a significant contribution from the aquaporins. However, changes in lipids could also contribute to this stability, either directly or indirectly. The molecular driving forces stabilizing the vesicles should be defined by membrane proteins and conformational properties of lipid bilayers. The fact that NaCl treatments provided more stable vesicles indicates that the adaptive conformational properties of membranes should be a fruitful approach for generating vesicles suitable as carriers.
